# Early prognostic score for neurological outcome at 6 months after cardiac arrest treated with hypothermia

**DOI:** 10.1186/2197-425X-3-S1-A969

**Published:** 2015-10-01

**Authors:** E Graziani, G Zani, M Negri, S Mescolini, C Gecele, FD Baccarini, R Ragazzi, M Fusari

**Affiliations:** Santa Maria delle Croci Hospital, Anesthesia and Intensive Care, Ravenna, Italy; Sant'Anna Hospital, Anesthesia and Intensive Care, University of Ferrara, Ferrara, Italy

## Introduction

The diffusion of BLS (Basic Life Support) and ALS (Advanced Life Support) has improved survival in patients that experienced cardiac arrest (CA). In after-ROSC comatose patients Therapeutic Hypothermia (TH), lowering the core temperature for 24 hours, has been demonstrated to ameliorate neurological outcome, but unfortunately during that period it is not possible to evaluate patient's neurological status and estimate prognosis.

## Objectives

Primary aim of the study was to evaluate which physiological, clinical and laboratoristic parameters, during the first 24 hours of TH after cardiac arrest, are related to the neurological outcome at 6 months; secondary objective was to define an early predictive score.

## Methods

This was a retrospective study on patients admitted to the general ICU of Ravenna's Hospital in a 3 years period (2011-2013) with a diagnosis of cardiac arrest and treated with TH, consisted in lowering core temperature to 32-34°C during the first 24 hours. Patients' physiological, clinical and laboratoristic data were hourly recorded by medical records. Inhospital mortality and neurological outcome at 6 months were also evaluated. A multivariate analysis was performed to detect which parameters were related to neurological outcome, defined as no or mild disabilities, moderate disabilities, severe disabilities or death. p < 0.05 was taken to be significant. A multivariate logistic regression was used to construct a predictive score for neurological outcome at 6 months.

## Results

In the analyzed period 47 patients were admitted to our ICU with a diagnosis of CA and 27 of them were finally enrolled because treated with TH. 21 patients had an out of hospital CA, with a mortality rate of 24%; 6 patients experienced an inhospital CA and the mortality rate was 33%. 10 patients had no or mild disabilities, 6 developed moderate disabilities, 5 resulted with severe disabilities and 7 dead. A worst neurological outcome at 6 months was related in the first 24 hours to: inhospital cardiac arrest (p < 0.05), higher blood lactate level (p < 0.01), higher blood glucose variation (p < 0.05) and the need of insulin administration (p < 0.05). A score was given to each of these parameters, depending on the detected value. The total score was the sum of the individual scores. A bad neurological outcome at 6 months was expressed by a total score ≥3, with a 99.5% of specificity and an AUC of 0.78.

## Conclusions

In patients treated with hypothermia after cardiac arrest, the finding of inhospital cardiac arrest, high blood lactate, high variation of blood glucose and the need of insulin administration during the first 24 hours is related to a worst neurological outcome at 6 months; a score that takes these parameters into account seems to early predict a bad neurological outcome, however larger, prospective and multicentre studies are needed to validate it.
